# Non-coaxial deformation of foreland basement involved in a fold-and-thrust belt: a strain partitioning approach to the Eastern Variscan orogen

**DOI:** 10.1038/s41598-023-35400-4

**Published:** 2023-05-19

**Authors:** L. Mareček, R. Melichar, J. Černý, P. Schnabl, K. Hrdličková, D. Buriánek

**Affiliations:** 1 Department of Geological Sciences, Faculty of Science, Masaryk University, Brno, Czech Republic; 2 Institute of Geology of the Czech Academy of Sciences, Rozvojová 269, 165 00 Prague-Lysolaje, Czech Republic; 3 Helmholtz-Zentrum Dresden-Rossendorf, Helmholtz-Institute Freiberg for Resource Technology, Freiberg, Germany; 4 Czech Geological Survey, Klárov 3, 118 21 Prague 1, Czech Republic

**Keywords:** Solid Earth sciences, Geology, Palaeomagnetism, Tectonics, Structural geology

## Abstract

The general SW–NE course of the Variscan orogen in Europe is abruptly bent to the N–S course at its eastern margin, where an oblique convergence occurred. The main suture in this part of the Variscan orogenic belt is called the Moldanubian Thrust, characterized by a dominant dextral strike-slip kinematics and a minor thrust component. The deep level of erosion and the good exposure of this structure allowed us to study the mechanisms of oblique convergence and the incorporation of the foreland basement into the orogenic belt. The combination of small-scale structures with the anisotropy of magnetic susceptibility studies allowed the recognition of two deformations in the studied rocks: dextral simple shearing and drag folding. Due to oblique convergence, the deformations induced by this mechanism were non-coaxial; therefore, their contributions can be easily distinguished. Finally, an overturned, almost recumbent large-scale synformal fold structure in the footwall and an antiformal structure in the hanging wall of the Moldanubian Thrust were formed. These two folds can be interpreted as structures formed by dragging along the Moldanubian Thrust. The previously described sinistral simple shearing in the upper limb of the synform resulted from the original dextral strike-slip shearing, which was overturned during progressive deformation.

## Introduction

Collisional orogenic belts are strongly thrusted and folded^1^ as a result of the convergent movements of continental tectonic plates. Two main mechanisms may have been involved during convergent orogenic movements. Most frequently, a simple model of only thrusting is considered^2^, while the more complex model of thrusting with overturning (as in Bhutan Himalaya^3^) has been described less often. The former model assumes a strongly inhomogeneous strain by simple shearing resulting in coaxially oriented structures^4^, while the latter model inevitably assumes a combination of coeval inhomogeneous deformations by simple shearing and bending^5,6^. The direction of shearing defines the simple shear geometry, while the geometry of bending deformation is determined according to the intersection of lithologic and shear planes (i.e., fold axes^7^). If the direction of thrusting is perpendicular to the orogenic front, these different deformations are coaxially superimposed on each other, and it can be difficult to distinguish them. Whereas in the case of oblique collision when the direction of thrusting is almost parallel to the orogenic front, both geometries are non-coaxial. This produces a polymodal pattern of structures, which allows for successful strain partitioning^8,9^ (see the chapter *Oblique thrusting orogens in the world* in the supplementary information). So far, oblique convergence has been studied in detail mainly in relation to recent collisional orogens and in the upper crustal levels^8^. However, there is no known detailed description of oblique thrusting from deeper crustal levels. This study intends to fill this gap in structural geology knowledge.

Differentiation of the various deformations under brittle-ductile to ductile conditions cannot be done without determining the orientation of mineral grains. However, direct observations of rock deformation are usually missing. In such cases, the anisotropy of magnetic susceptibility method (AMS) can be used to determine the preferred orientation of mineral grains reflecting the strain directions. In this way, the AMS method can be proven effective in the separation and determination of individual deformations, as it allows one not only to determine strain directions as a reflection of the preferred orientation of mineral grains, but also to quantify the degree of anisotropy and, as a result, the strain magnitude^6,10^. The usefulness of the method was previously demonstrated in deformed granitoid^10,11^ as well as in nondeformed magmatic rocks with magmatic fabrics^12,13^.

In this paper, we focus on the explanation of coeval sinistral shears present in the predominantly dextral shearing environment. We also attempt to differentiate between external and internal rotations as a unique case of non-coaxial development of various kinds of deformations in the most eastern Variscan orogen along the Moldanubian Thrust, where oblique thrusting occurred. For such tasks, we used the anisotropy of magnetic susceptibility (AMS) method reflecting internal fabrics of rocks in combination with the conventional tectonic data studies.

### Geological setting

**Figure 1:**
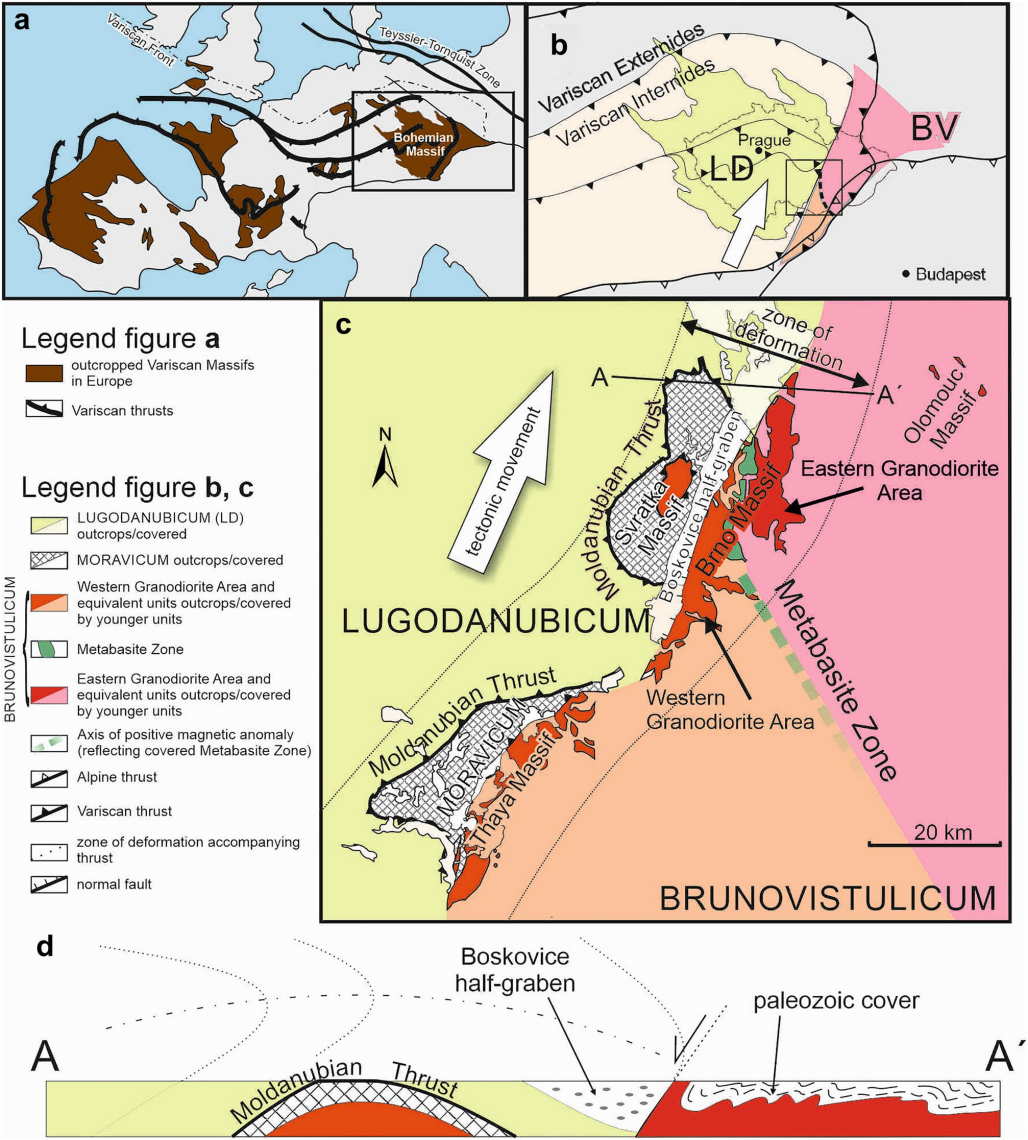
Tectonic setting of the study area within the European Variscan orogenic belt: (**a**) Location of the Bohemian Massif in the context of the Variscan orogenic belt in Europe (modified after^26^; (**b**) Oblique thrusting of Lugodanubicum (LD) over Brunovistulian unit (BU) occurred in the Eastern part of the Bohemian Massif (modified after^27^; (**c**) The position of the Brno Massif and its relationship to the Moldanubian Thrust and accompanying zone of deformation (modified after^27^; (**d**) Simplified profile through the contact of Lugodanubicum and Brunovistulian unit (modified after^28^). The figure was created using Corel Draw 2020 software (https://www.coreldraw.com/en/product/coreldraw/).

The European Variscides form an orogenic belt occurring in several erosional windows in southwestern, western, and central Europe. The central European (i.e., eastern) part of the Variscan belt, known as the Bohemian Massif (Fig. 1a), has a profound erosion level that allows us to study deep crustal structures. Moreover, the Variscan orogeny on the eastern margin of the Bohemian Massif ended with a lateral collision of two main plates involving successive deformation phases^14,15^. One of the colliding plates was the early Variscan mega-unit of Lugodanubicum^16^, consisting of several regional units from the Moldanubicum in the south to the Lugicum in the north. During the Variscan collision, the Lugodanubian plate overthrust lower plate, named Brunovistulicum (also referred to as the Brunovistulian unit), which can be considered as a foreland of the Variscan orogen^14,15^ (see Fig. 1b and the chapter *Brief insight to tectonic history of the eastern margin of Central European Variscides* in the supplementary information). The suture between these two tectonic units is broadly known as the Moldanubian Thrust, and it is accompanied by a wide zone of strong simple shear strain along the main thrust surface, which strikes SSW–NNE and dipping gently W in general^17^ (Fig. 1c). The dominant sense of tectonic movement along this thrust zone is top-to-the NNE, i.e., a dextral strike slip with a small component of thrusting^18–20^, which is evidenced by the formation of an overturned antiformal fold in the Lugodanubian unit above the thrust^21–23^ (Fig. 1d). Although the deformation in the upper plate (i. e. Lugodanubicum) has been intensely studied (see^24,25^ for review), the information on the deformation mechanism in the lower plate is very limited^14,19^. Moreover, the study area provides a specific case of foreland deformation in the thrust footwall of the Variscan orogen with oblique thrusting nearly parallel to the deformation front^24,25^.

The footwall of Moldanubian Thrust is represented by the Brunovistulian unit (also referred to as Brunovistulicum). The Brunovistulian unit (BU) mainly consists of a Cadomian basement (655 ± 3 Ma^29,30^ best outcropped in the Brno Massif (Fig. 1c). A zone of highly strained rock that forms a relatively thin sheet in the western part of the BU is usually defined as a separate unit called the Moravicum^14,31^. The typical structural pattern in the Moravicum and adjacent deformation zone is an inverse metamorphic sequence (i.e., the degree of metamorphosis increases upward) with flat foliation and NNE-trending stretching lineation^14,19^.

**Figure 2:**
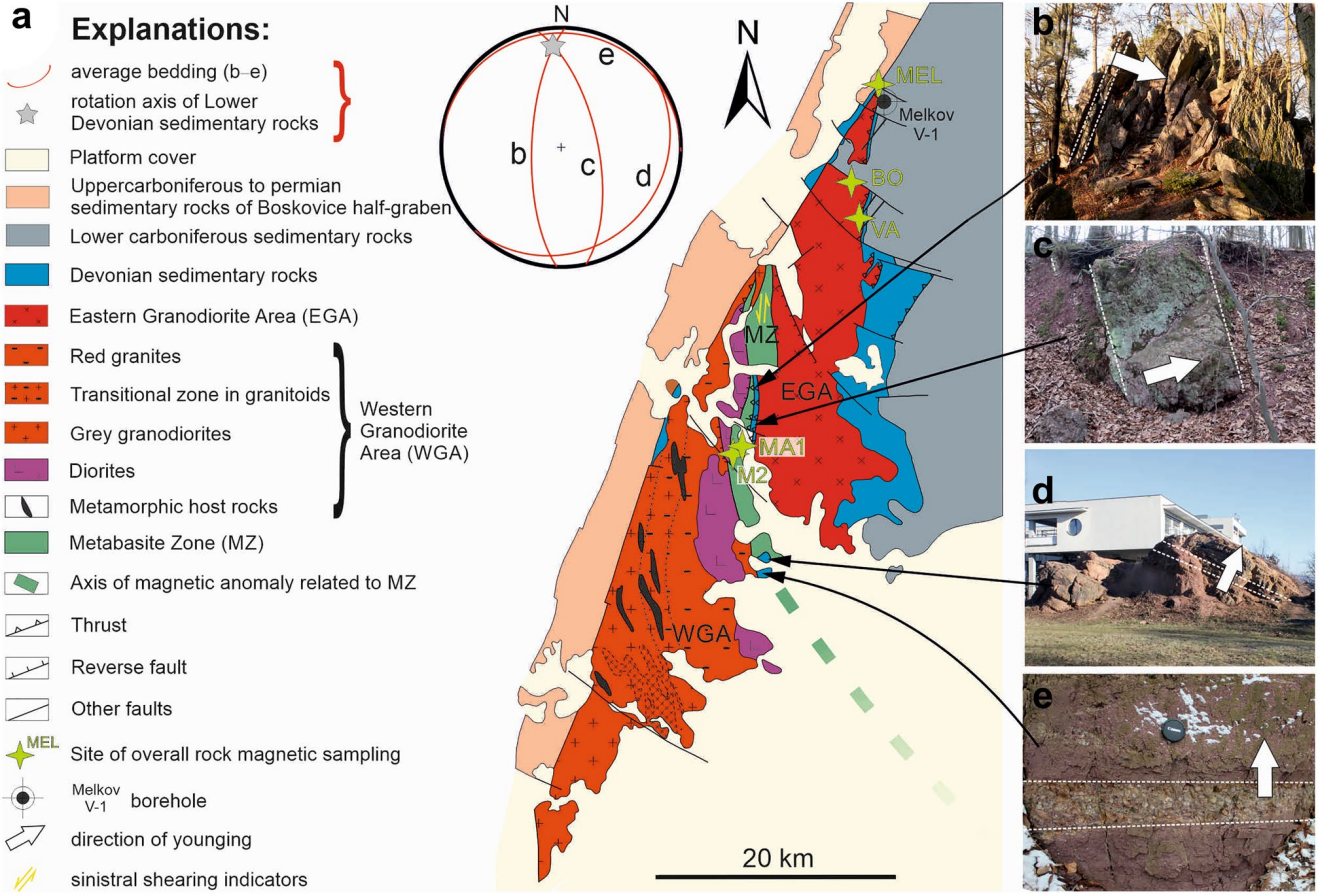
Structural subunits of the Brno Massif (**a**) and orientation of bedding in a bent tectonic sheet of Devonian sequence incorporated to Neoproterozoic granitoid rocks (b–e) where white arrows indicate facing of the beds; (**a**) Zoning of granite types in the Western Granodiorite Area according to^27,32^; (**b**) Overturned beds dipping to the W in the North (Babí lom locality); (**c**) steep beds in the central part (Lelekovice); (**d**, **e**) slightly inclined to subhorizontal beds in the South (d—Žlutý kopec; e—Červený kopec). The figure was created using Corel Draw 2020 software (https://www.coreldraw.com/en/product/coreldraw/).

The Brunovistulian basement that outcrop in the studied Brno Massif consists of three areas representing old terranes (Figs. 1b, 2). Two Cadomian granodiorite units descriptively termed the Western and the Eastern Granodiorite areas are separated by the pre-Cadomian Metabasite Zone in the middle^20,32^ (Fig. 2).

The Metabasite Zone (MZ) consists of the basic volcanic rocks metamorphosed under greenschist-facies conditions^32^. The zone strikes N-S and extends southeastward, beneath the post-Variscan sedimentary cover, where it is marked by a high positive magnetic anomaly^33,34^. The rhyolite dikes transecting the MZ were dated at 725 ± 15 Ma^35^, therefore, the metabasalts of the MZ are older.

The Western Granodiorite Area (WGA) contains mostly S-type granitoids^32^ and large relics of metamorphic host rocks (paragneiss, migmatite, diorite, calc-silicate rocks). The host rocks, as well as diverse types of granodiorites, form N–S elongated belts. The elongation turns NW–SE in the southern parts of the belts following the course of the MZ^32,36^. The age of the host diorite rock was determined at 655 ± 3 Ma^30^, while the radiometric age of the granitoids is 601 ± 3 Ma^29^.

The Eastern Granodiorite Area (EGA) is relatively homogeneous in its rock composition mainly consisting of I-type biotite to amphibole-biotite granodiorites^37^ of the Neoproterozoic age (~ 601–590 Ma^38^).

The Brunovistulian unit basement is autochthonously covered by Devonian basal conglomerates and limestones on which lies a (para)autochthonous to an allochthonous accretionary wedge of Lower Carboniferous flysch rocks^39^. Both the Brunovistulian basement and its overlying sedimentary cover were affected by oblique thrusting, folding, and low-temperature metamorphism during the Variscan orogeny.

The east-vergent folds in the sediments have subhorizontal fold axes that plunge slightly to the N in the area of interest^40^. The advancing compression led to the incorporation of the western part of the Brunovistulian basement into the thrusted and folded sedimentary complexes^20,41^. It resulted in a large, overturned syncline on the eastern side of the Brno Massif close to the Moldanubian Thrust (Fig. 1d). The overturning of the rock sequence in the upper (western) limb of the syncline was documented by the Melkov V-1 borehole (Fig. 2), in which the Devonian, and beneath it, the Carboniferous sedimentary sequence was found tectonically overthrust by the older Neoproterozoic granitoids of the Brno Massif^42^.

Similarly, to the eastern side of the Brno Massif, it is possible to trace tectonic sheets of the lower Devonian sedimentary rocks incorporated between large sheets of the Brno Massif. They are also bent into a large, overturned drag fold in the footwall of the Moldanubian Thrust together with surrounding granitoid rocks (see Fig. 2; also see^40^). These fold-related deformations correspond to the thrust-folding of the Variscan accretionary wedge, as evidenced by the identical orientation of the fold axes and axial surfaces in the Devonian to Lower Carboniferous sedimentary rocks in the east^40^.

Together with folding, a simultaneous inhomogeneous deformation occurred and resulted in the formation of brittle-ductile shear zones gently to moderately dipping W^41,43^, the orientation of which corresponds to the axial surfaces of the aforementioned large folds. In granitoids, these shear zones are marked by low-temperature metamorphism evidenced by recrystallization under greenschist-facies conditions, i.e., epidotization, chloritization, and sericitization^32^. Furthermore, these shear zones are also accompanied by an SSW–NNE trending stretching lineation with a dextral top-to-NNE sense of movement^44^, which is documented by the presence of a dextral strike-slip component adjacent to the Moldanubian Thrust^14,19^. For a long time, only dextral senses of movement responding to the Moldanubian Thrust were considered, even after sinistral movements in the northernmost MZ had already been already recognized^45^. Later findings of asymmetric porphyroclast systems and S-C fabric in metabasalts^46^ confirmed the presence of a sinistral sense of shearing along this N–S zone (MZ, see Fig. 2). However, the tectonic models of the eastern margin of the Bohemian Massif published so far^14,31^ cannot explain these sinistral shears.

The post-Devonian age of all described deformations associated with Moldanubian Thrust is evidenced by (1) tectonic sheets of Devonian limestone incorporated into the Cadomian basement^40,41^ and (2) by the identical low-temperature metamorphism of Devonian cover determined using the conodont alteration index to 300 °C^47^ as well as the granitoids of the Brno Massif evidenced by recrystallization under the greenschist facies^32^. Given these facts with the age of the oldest rocks in the Boskovice half-graben that are not folded or metamorphosed by these processes (i.e., the latest Carboniferous—Gzhelian^48^), the deformations correspond to Variscan orogeny.

## Results

### Petrography and deformational fabrics

**Figure 3:**
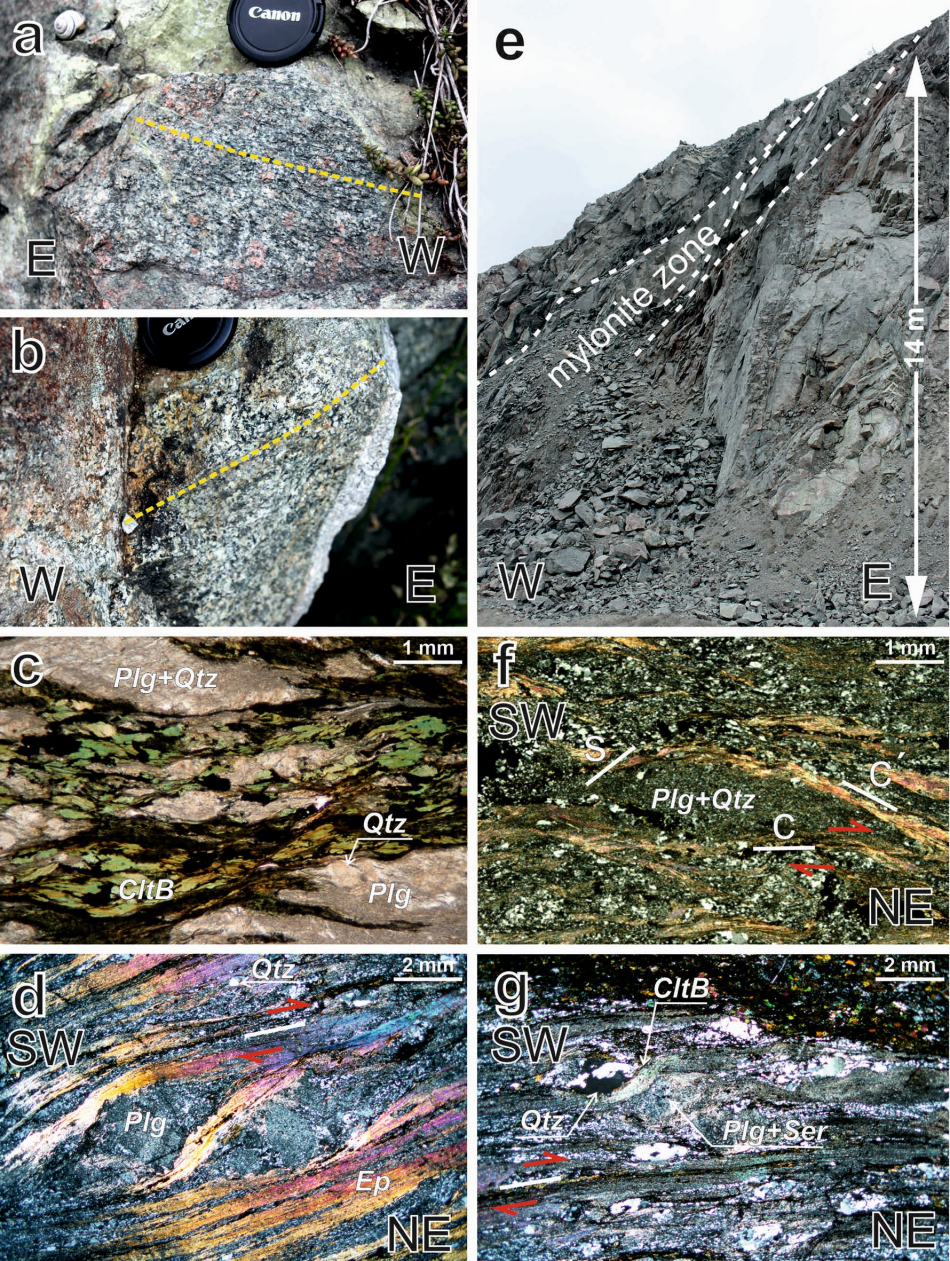
Dominating structures recognized in rocks of the Brno Massif: small-scale strain foliation defined by the preferred orientation of mineral grains in (**a**) the EGA, and (**b**) in diorites of the WGA; (**c**, **d**, **f**, **g**) thin-section photomicrographs of foliated granitoids from the WGA (polarized light) with shearing indicators (e.g. an S–C–C´ structure); (**e**) slightly foliated granites with a small zone of strong foliation in the northern part of the Brno Massif (Doubravice quarry).

The magmatic origin of the rocks of the Brno Massif is evidenced by the relics of mafic enclaves, xenoliths, and zoned euhedral plagioclase grains. The magmatic fabrics are inhomogeneously overprinted by later deformation (Fig. 3a,b). The ductile shear zones are related to the formation of mylonites (Fig. 3d). The deformed rocks of the Brno Massif have the following deformational characteristics:

The strained biotite flakes are bent and concentrated into more or less parallel bands; chloritization of this biotite was often observed (Fig. 3c). Feldspar grains are twinned; the twins are fine, multiple, and incoherent, which implies their origin in deformation. Highly strained feldspar grains are fractured to small grains and/or replaced by newly formed sericite, epidote, and albite. Such aggregates (formerly feldspars) and chloritized biotite are stretched and form a more or less visible foliation and lineation of rocks. In this way, granodiorite is changed to metagranodiorite, greenish mylonite, or chloritic phyllonite with small “eyes” of quartz grains situated in a matrix of newly crystallized phyllosilicates. The mylonite zones are 0.1 m to tens of meters thick. The largest mylonite zones were found in the NE part of the Brno Massif. Their foliation dips to the W–NW, respecting the general dip direction of the Moldanubian Thrust. The simple shear deformation is documented by dextral thrusting asymmetric structures, such as σ-porphyroclast systems (quartz grains) and/or S–C–C´ structures (Fig. 3c), and by discrete shear zones in granodiorite (Fig. 3d).

### Rock magnetic studies and geometry of AMS fabrics

**Figure 4:**
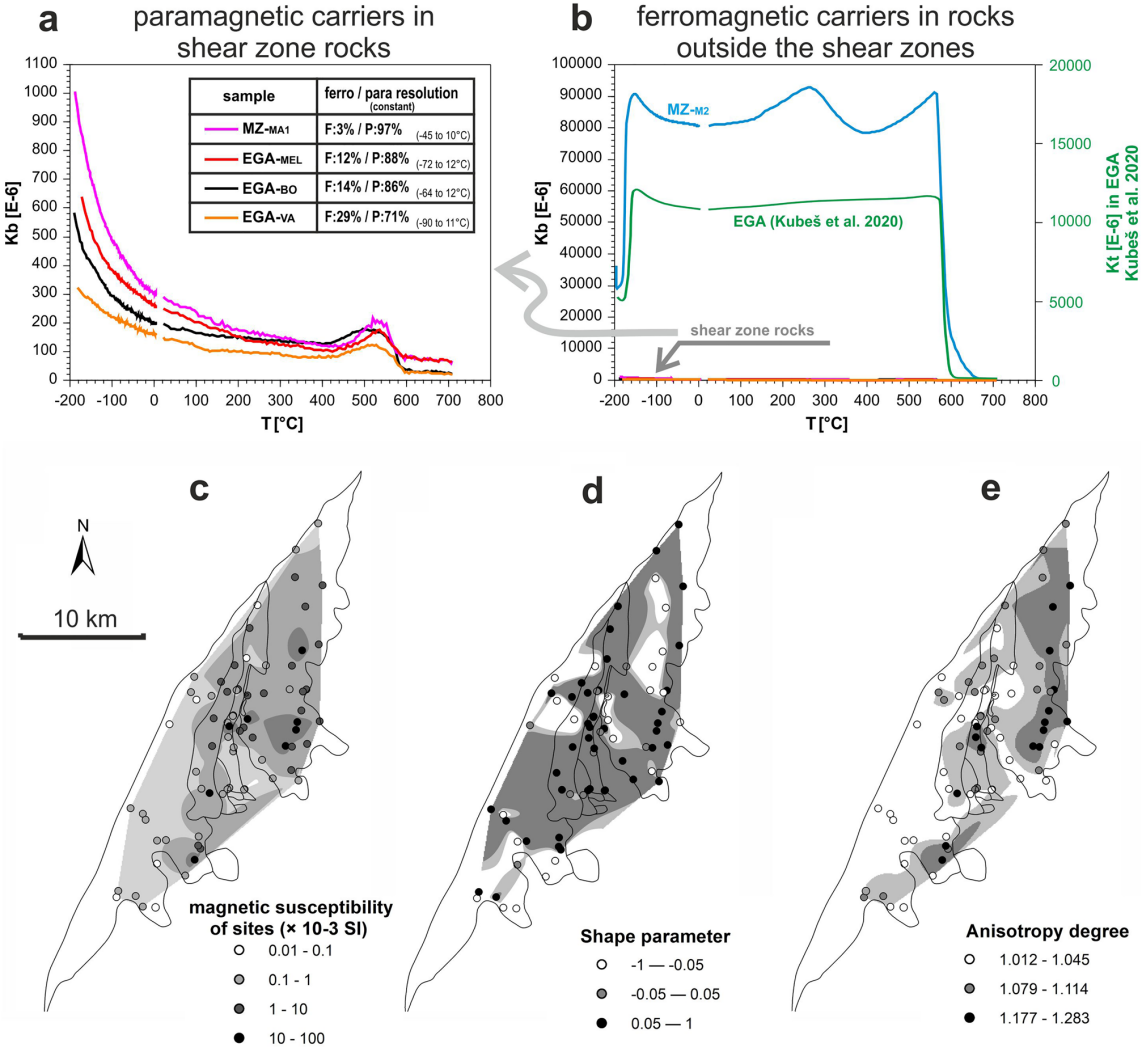
Thermomagnetic curves and AMS parameters of rocks of the Brno Massif: (**a**) Heating curves of strained rocks of the Brno Massif; the heating curves have a descending character up to ~ 250 °C, reflecting that paramagnetic minerals are the dominant minerals controlling AMS at room temperature. The table shows a semi-quantitative calculation (within the interval) of magnetic susceptibility carriers; (**b**) Thermomagnetic curves of non-strained granodiorites (according to^34^ and metabasites; (**c**) Mean magnetic susceptibility Kmean; (**d**) Shape parameter T (white–prolate; gray–triaxial; dark gray–oblate shape); (**e**) Anisotropy degree P (the darker the gray color, the stronger the anisotropy). The isolines in (**c**), (**d**), and (**e**) were created by the natural neighbor interpolation method with a cell size of 94.3 m. Explanation: MZ—Metabasite Zone, EGA—Eastern Granodiorite Area with localities BO – Boskovice, VA—Valchov, MEL—Melkov. Parts of figure (**c**, **d**, **e**) were created using ArcGIS 10.2 software (URL: https://www.esri.com/en-us/arcgis/products/arcgis-desktop/overview)). The figure was compiled using Corel Draw 2020 software (URL: https://www.coreldraw.com/en/product/coreldraw/).

Thermomagnetic analyzes of granodiorites and metabasites revealed that non-strained rocks in the Brno Massif generally contain considerable amounts of magnetite, indicated by the Verwey transition at − 150 °C and Curie temperature at 585 °C (Fig. 4b). They indicate the predominance of multidomain (MD) magnetite grains^49^. In general, the magnetic susceptibility of granodiorite from the EGA is higher by at least two orders of magnitude than that of deformed granodiorites from the shear zones (compare Fig. 4a,b). The magnetic susceptibilities of rocks in the eastern part of the Brno Massif are generally controlled by the formation of metasomatic magnetite^34^. However, the low magnetic anomalies coinciding with shear zones result from the replacement of secondary magnetite by martite^18^. This is confirmed by the temperature dependence of the magnetic susceptibility analysis in strained rocks, showing a continuous decrease in magnetic susceptibility with an increasing temperature to ~ 450 °C (Fig. 4a). This indicates that the AMS of the strained rocks is driven by paramagnetic minerals. The sudden increase in magnetic susceptibility at temperatures above ~ 450 °C indicates the presence of single-domain magnetite (SD), which is supported by IRM curves (Supplementary Fig. S1). However, the resolution between ferromagnetic and paramagnetic contributors to magnetic susceptibility has shown a complete predominance of paramagnetic minerals as indicated by the hyperbolic character of all curves up to room temperature and beyond from the sheared zone samples (Fig. 4a). Therefore, the room-temperature AMS from the shear zones reflects paramagnetic minerals. In addition, the increase of magnetic susceptibility around 250 °C in more or less unstrained metabasites (MZ-M2) is probably caused by goethite. However, its contribution at room temperature is negligible^50^.

The magnetic susceptibility values of the rock range from 2.02 × 10^–5^ to 1.16 × 10^–1^ SI. The highest values of magnetic susceptibility were found in the EGA, corresponding to non-deformed I-type granitoids, whereas the WGA with S-type granitoids revealed the lowest values (Fig. 4c). Highly deformed granodiorites form the EGA are typical of low magnetic susceptibility^18^. The mafic rocks of the WGA (i.e., diorite) and the MZ show naturally high magnetic susceptibilities.

The values of the shape parameters extend widely from − 0.96 to 0.98, however, most of the samples show oblate magnetic fabrics (Fig. 4d). The values of the anisotropy degree (*P*) in the measured samples range from 1.005 to 1.485. In the Brno Massif, *P* generally increases towards NE and the highest *P* values are displayed by rocks in the eastern part of the EGA even though there are a few isolated local maxima of *P* values in some parts of the MZ, which has the highest vulnerability to subsequent deformation (Fig. 4e). The highest *P* values correspond to a macroscopic observation of heavily foliated granitoids. An increase in the strain from SW to NE was also observed in the microstructures of the granodiorites.

### Orientation of mesoscopic and AMS fabrics and their distribution

**Figure 5:**
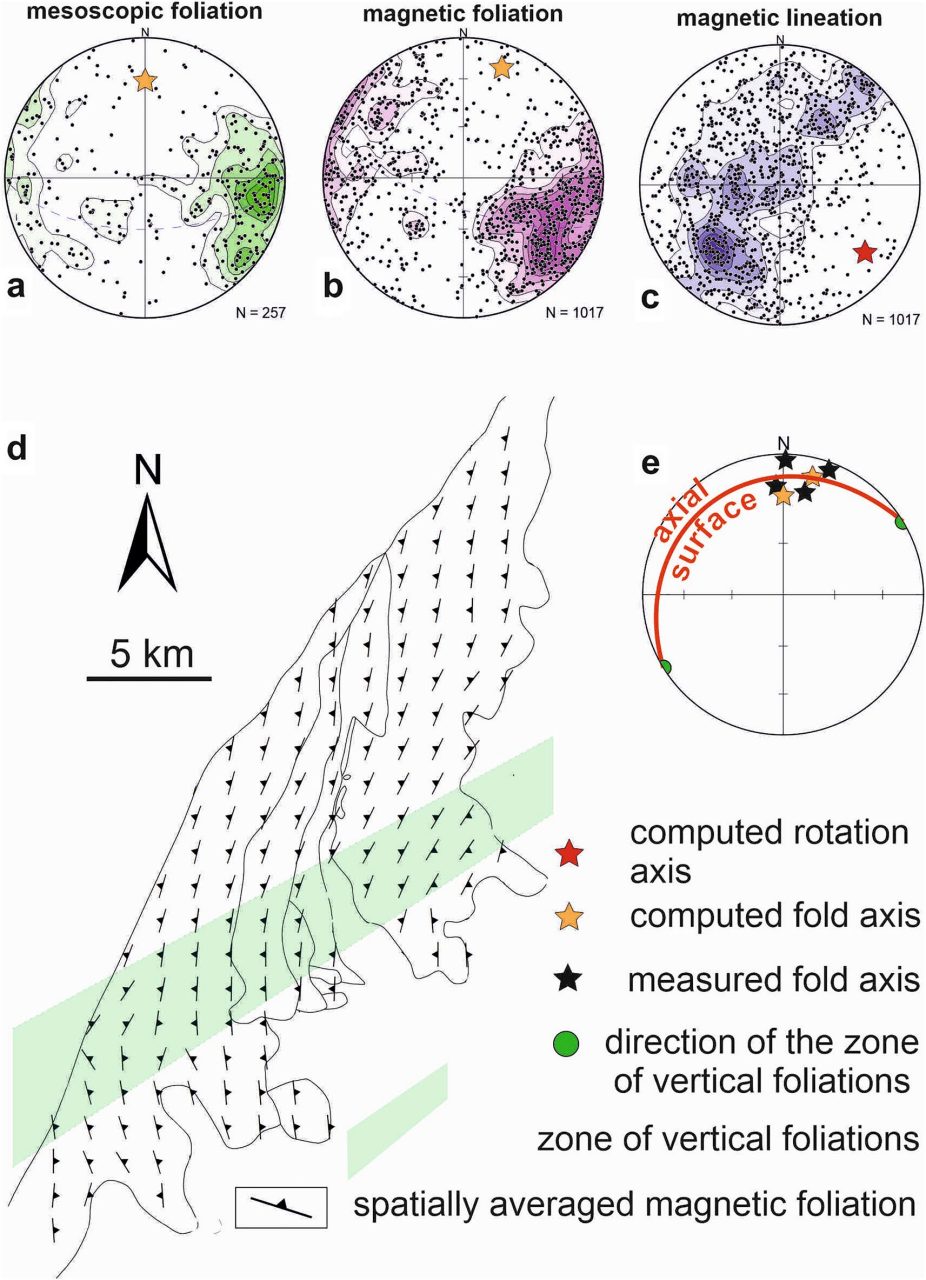
Orientations of structural elements in stereoplots (**a**–**c**). The computed fold axes from the foliations (**a**, **b**) are indicated by yellow stars, while the measured fold axes in Devonian sedimentary rocks adapted from^44^ are indicated by black stars (**a**); the computed rotation axis from the magnetic lineation (**c**) is indicated by a red star. Diagrams (**a**, **b**, **c**) are in equal-area Schmidt projection on the lower hemisphere, contour lines with interval 2 × standard deviation. Spatially averaged map of magnetic and mesoscopic foliations with the zone of vertical foliations indicating the axial trace (**d**) and the geometric relationship of fold axes, axial trace and fold axial surface (**e**). Part of figure (**d**) was created using ArcGIS 10.2 software (URL: https://www.esri.com/en-us/arcgis/products/arcgis-desktop/overview).). The figure was compiled using Corel Draw 2020 software (URL: https://www.coreldraw.com/en/product/coreldraw/).

The results of the structural analysis revealed a significant maximum in the poles of the mesoscopic foliation striking SSW–NNE with a slight transition to N–S and occasionally NNW–SSE (Fig. 5a). The contours of magnetic foliations form almost the same pattern as those of mesoscopic foliations in a stereographic projection (compare in Fig. 5 and Supplementary Fig. S3). This means that they presumably show a straightforward correlation, and hence also the same deformation fabric. The variation in the orientations of the mesoscopic and magnetic foliation poles allowed us to calculate the orientation of the fold axis using a principal direction analysis (yellow stars in Fig. 5a,b). The resulting fold axes plunge to N–NNE, which agrees with the orientations of the mesoscopic fold axes obtained from the Devonian sedimentary rocks (black stars in Fig. 5e and gray stars in Fig. 2). Magnetic lineations lie in a wide girdle with the rotation axis trending SE (red star in Fig. 5c), which significantly differs from the trends of fold axes directly measured or calculated from the foliations (compare Fig. 5a–c).

The tectonic map of mesoscopic and magnetic foliations averaged for individual sites shows a very inhomogeneous distribution of their strikes (Supplementary Fig. S2), which is also consistent with the imperfectly developed preferred orientation of both kinds of foliation (Fig. 5a,b). However, it can be observed that most of the foliation planes in the northern part of the Brno Massif strike NNE–SSW to N–S and then dip to the W, while the foliations in the southern part strike NW–SE and then dip to the NE (Supplementary Fig. S2). These relationships are visible in a tectonic map with spatially averaged orientations of magnetic and mesoscopic foliations (Fig. 5d). These averaged directional data show a zone of westerly-dipping foliations in the northern part of the Brno Massif and easterly-dipping foliations in its southern part. The change in the direction of the dip takes place in a zone of vertical foliation indicating the existence of an overturned, gently inclined to recumbent fold as a large-scale structure in the study area. The zone of vertical foliation reflects that the trace of fold axial surface trends approximately in the SW–NE direction. Knowing the trend of the axial trace and the fold axis, the axial surface of the fold can be computed. It dips gently to the NW (Fig. 5e).

## Discussion

Most of the fabrics in the Brno Massif show deformational fabrics that resulted from a strong overprint during Moldanubian Thrust activity. Arguments for the deformational origin of the main part of the fabrics are the following: (1) A high degree of anisotropy in the NNW part of the Brno Massif (Fig. 4e). (2) The presence of deformational fabrics in thin sections and on a mesoscopic scale and almost no visible primary magmatic structures (Fig. 3). (3) Presence of the greenschist facies metamorphism in all rocks of Brno Massif. The temperature condition of the deformation corresponds to the greenschist metamorphism, which agrees with the described mineralogy and chlorite and/or epidote fillings of the brittle fractures we found. Furthermore, the relics of quartz grains indicate that the temperature during the deformation processes did not reach 300 °C^51^. These temperature characteristics of the deformations correspond to observations made by^32^, i.e., recrystallization under the greenschist facies, and to the tectonic affection of the Devonian sedimentary rocks based on the conodont alteration index to 300 °C^47^. (4) A major part of the recognized fabrics across granitoids in the Brno Massif maintain regional-scale orientations and preserve structural patterns in the surrounding non-magmatic rock. This is not likely to be related to primary magmatic fabrics^52^. (5) Most importantly, the northern part of the Brno massive lies directly beneath the Moldanubian Thrust, one of the most significant tectonic structures in the Variscan orogeny representing the collision of the Lugodanubian plate with the Brunovistulian unit in the easternmost part of European Variscides. Strong deformation is also evidenced by tectonic sheets of the Devonian sequence as well as by outcropping tectonic relics of Brno Massif granitoids deep in the Moravian Karst in the east^53^.

**Figure 6:**
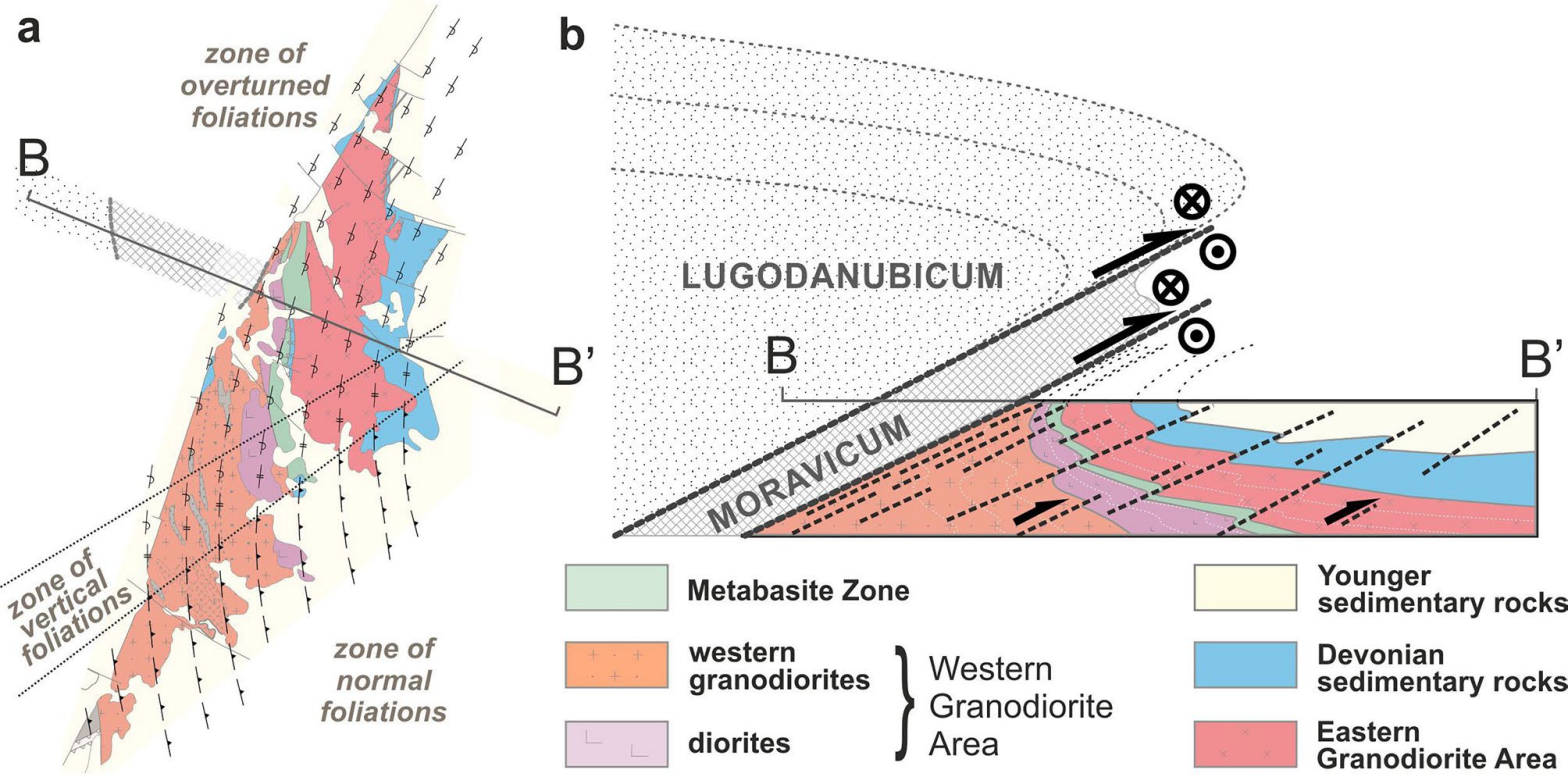
Structural model of the Brno Massif immediately after thrusting (i.e. in the Upper Carboniferous, before the formation of the Boskovice half-graben): (**a**) schematic trend map of foliation strikes and their relation to lithological zones^27,32^; note that the NW part is characterized by overturned foliation; and (**b**) resulting schematic cross-section with the synclinal structure of the Brno Massif in the footwall of the Moldanubian Thrust and an antiformal structure of the Lugodanubicum in the hanging wall, which was sketched previously by several authors^21–23^. The black arrows show a minor component of the Variscan thrusting, while the main dextral tectonic movement is indicated by arrowhead/feather marks. The figure was compiled using Corel Draw 2020 software (URL: https://www.coreldraw.com/en/product/coreldraw/).

To understand the complicated situation on the eastern margin of the Variscan orogen, we created a simplified schematic map of strikes of the recognized foliations (Fig. 6a), which provides a fundamental view of the structure of the Brno Massif. The strike lines of averaged foliations form a bend from NNE-SSW strike directions in the north to NW–SE in the south, and the corresponding flexion can also be seen by the flexure of the tectonic sheet of Lower Devonian sedimentary rocks inside the Brno Massif, in lithological zoning of the Brno Massif such as granite suite zones^32^, in zones of host rocks such as calc-silicate rocks, migmatite and diorite in the WGA^36^, and especially in the bending of the MZ, the continuation of which to the SE beneath the sedimentary cover is evidenced by a high positive magnetic anomaly (see Fig. 2). The described bending of the strike lines can be explained by the large-scale overturned, gently inclined to recumbent fold, with the fold axis plunging approximately to the north and the axial surface dipping to the NW. This indicates that the upper limb crops out in the north while the lower limb lies in the south and the transition area between the limbs across the zone of vertical foliation precisely coincides with the bend in the strike lines as well as with the hinge zone of a large-scale drag fold directly beneath the Moldanubian Thrust, i.e., megathrust between two pre-Variscan units representing previously two independent tectonic plates^23^.

The facing of the fold, determined by the Devonian to Lower Carboniferous sedimentary complex incorporated in the structure, proves that this large-scale fold is a syncline (sensu stricto), with younger rocks in its core. Therefore, the lower (i.e., southern) limb is normal, while the upper (northern) limb is overturned. Such a structure with an inclined fold axis allowed us to use the down-plunge method^54^ for the reconstruction of a regional tectonic model (Fig. 6b).

The NW-dipping axial plane is more or less parallel to the orientation of shear zones, which have been previously described^41,43^. These shear zones disrupt the entire region, but the overturned fold limb adjacent to the Moldanubian Thrust is the most affected, i.e., the deformation is localized along the Moldanubian Thrust (Fig. 6). The Variscan age of the described deformations also corresponds to this model.

**Figure 7:**
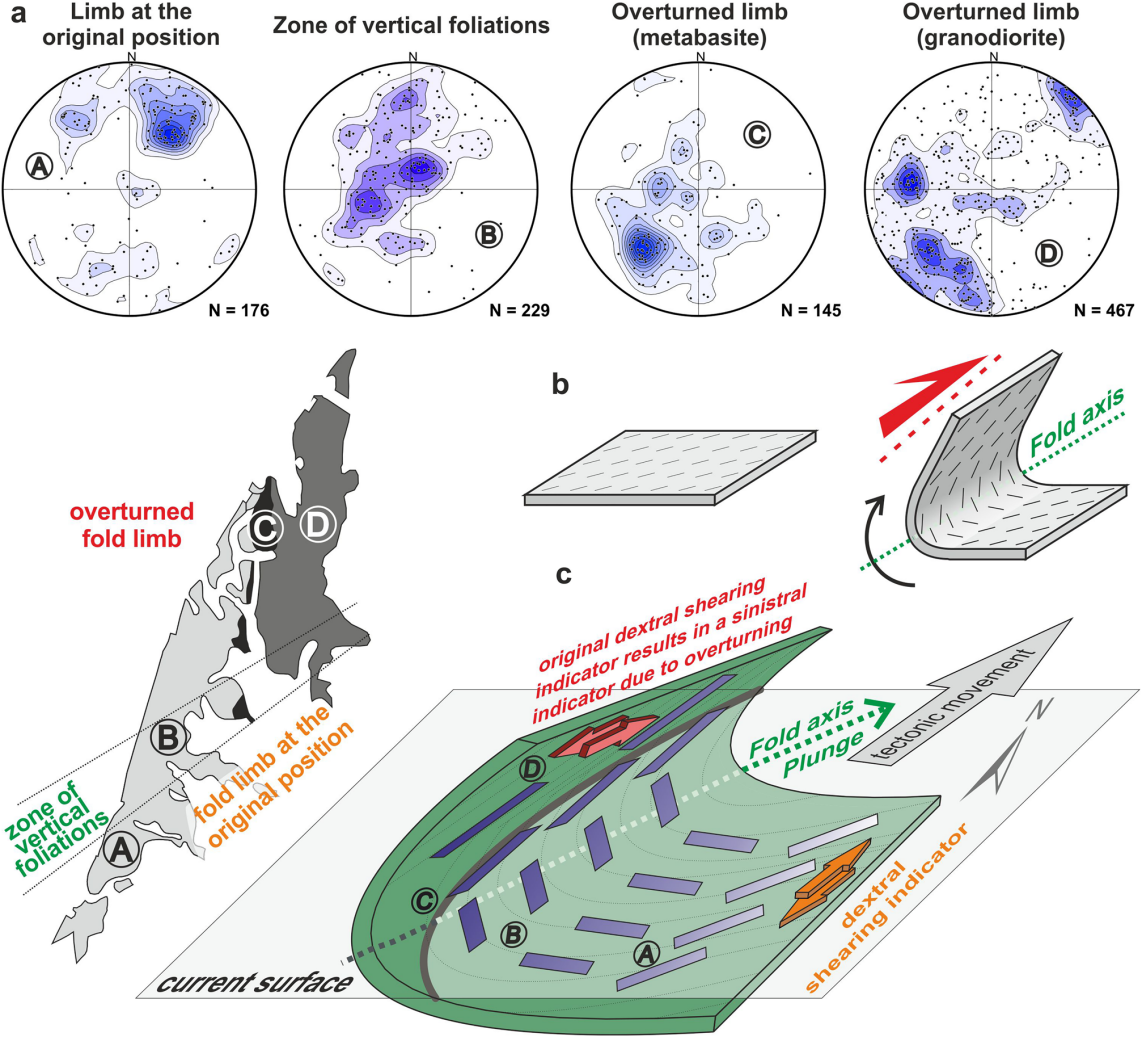
The distribution of magnetic lineations on a schematic map of the Brno Massif^27^ and their trends in a 3D model explaining the 2D situation in the map: (**a**) different orientations of magnetic lineation in different parts of the Brno Massif, equal-area Schmidt projection on the lower hemisphere, density distribution contour lines with interval 2 × standard deviation; (**b**) Schematic origin of the deviated lineation orientation in the overturned limb of the drag fold in the footwall of the Moldanubian Thrust; and (**c**) final 3D scheme of a large-scale drag fold with its axis plunging northward in relation to the present erosional surface. The spatial distribution of magnetic lineations (red arrows) is projected on the fold surface. The figures (c, d, e) were created using ArcGIS 10.2 software (URL: https://www.esri.com/en-us/arcgis/products/arcgis-desktop/overview). The figure was compiled using Corel Draw 2020 software (URL: https://www.coreldraw.com/en/product/coreldraw/).

The key information necessary for understanding the mechanism of deformation is encrypted in the spatial distribution of magnetic lineations. The lower limb of the syncline is characterized by magnetic lineations that plunge to the NE at a moderate angle, whereas the lineations in the deformed overturned limb are subhorizontal or gently plunge to the SW. The transitional area between these two limbs, that is, the zone of vertical foliations, is represented by sub-vertical or very steep lineations (Fig. 7a). The NW part of the Brno Massif shows a strong overprint by an intensive deformation (Fig. 6b), which is manifested by (1) a high degree of anisotropy (Fig. 4e); (2) no visible primary magmatic structures; and (3) by the strong parallelism of planar structures such as mesoscopic anisotropy surfaces, AMS surfaces, boundaries of lithological zones, elongation of the MZ as well as host rock zones, and the course of the stratification in Devonian rocks. This higher strain intensity and high recrystallization under greenschist facies conditions indicate that this deformational fabric is younger than that of only slightly deformed granodiorite in the SE part of the Brno Massif, where primary magmatic fabrics are commonly present. Therefore, the fabric from the southern part of the Brno Massif in the normal limb with lineation plunging to the NE can be considered the oldest, and the fabric from the northern part in the overturned limb with lineations plunging SW is the consequence of higher strain produced by movement along the Moldanubian Thrust and should be considered younger.

**Figure 8:**
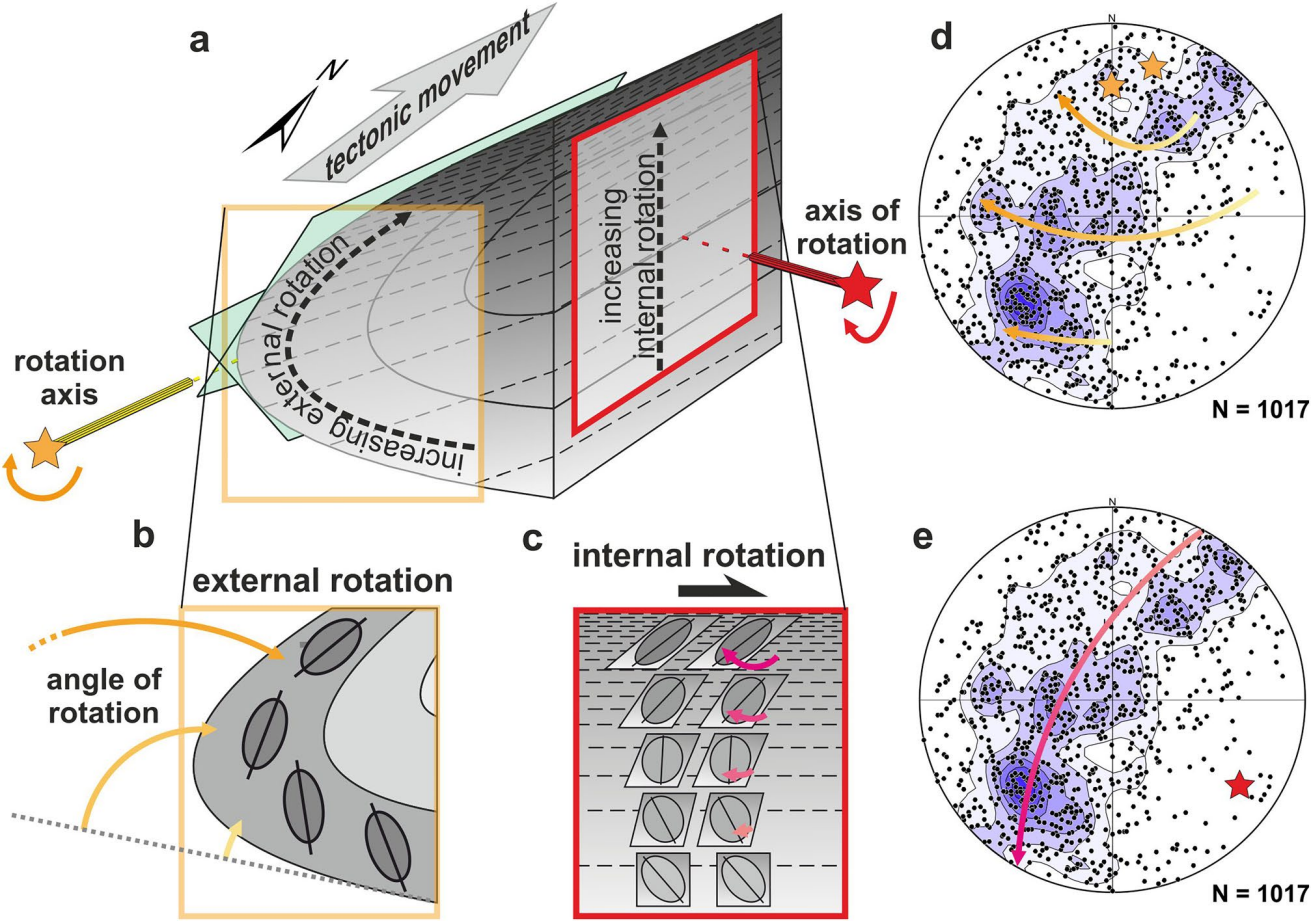
Deformations recognized in rocks of the Brno Massif. The whole structure results from a combination of two non-coaxial deformations (**a**): external rotation of the rock fabric caused by a bend (**b**) and internal rotation connected to simple shearing of the rock mass (**c**). These deformations can be recognized in the contour diagram of magnetic lineations: small circles indicate external rotation around the fold axis (**d**), and a wide girdle is produced by an internal rotation around its SE-plunging axis (**e**).

The magnetic lineations in the Brno Massif gradually change orientation from a plunge to the NE through a vertical position to a plunge to the SW (Fig. 7a: A to D). This change can be explained by internal rotation around an axis perpendicular to the direction of displacement (Fig. 8a,c,e), which was caused by dextral simple shearing with top to the NNE (Fig. 7b). The axis of the internal rotation during simple shear deformation is identical to the principal axis of the main magnetic lineation girdle (the red star in Figs. 5c and 8e). Analyzing the main girdle in more detail, it is possible to identify secondary transverse belts forming small circles. The corresponding rotation axis (the yellow stars in Fig. 8) is parallel to the large-scale fold axis computed from magnetic foliations and measured in the field (the yellow and black stars in Fig. 5). Consequently, we can associate the origin of these small circles with the external rotation of the upper limb during the formation of the large-scale fold identified in the map of the area (Fig. 8a,b,d). This way, the wide girdle of magnetic lineations (Fig. 8d,e) can be explained by a combination of two simultaneous rotations with transversely orientated axes of rotation (Fig. 8d vs. e; yellow stars vs. red stars).

Our results imply that the upper overturned limb of the large-scale fold exposed in the NW is the most strained part of the Brno Massif, and two different but simultaneous deformations: (1) the first was inhomogeneous simple shearing with the top to the NNE in both the whole rock mass and the more or less discrete shear zones (Fig. 8c), and (2) the second deformation was bending and associated overturning (Fig. 8b). These two deformations usually produce coaxial rotation when the crustal nappes are thrust over the foreland in a direction approximately perpendicular to the orogenic front; consequently, their individual effects are difficult to distinguish. In the study area, however, thrusting was subparallel to the frontal line. As such, it allowed us to distinguish between the two deformations and their effects. As a rule, while the axis of internal rotation during simple shearing lies in the shear plane and it is perpendicular to the movement vector, the axis of external rotation during bending in the shear zone coincides with the intersection of older anisotropy surfaces and shear planes irrespective of the movement vector^7^. In this way, the resulting structure of the Brno Massif can be explained by a combination of simultaneous simple shearing and bending.

The deformation zone along the Moldanubian Thrust consists of two opposing large drag folds—an anticline formed by highly metamorphosed rocks in the hanging wall^21,22^ and a syncline with rocks deformed under the low-temperature conditions described above. As a result, both fold structures can be explained as drag folds formed during movement along a major thrust (Moldanubian Thrust).

The previously described dextral shearing accompanying the Moldanubian Thrust, i.e., top to the NNE, was confirmed. However, the study of the described deformations also allowed us to explain the mysterious sinistral simple-shear kinematic indicators with top-to-the-South sense of movement. Their discovery only in the overturned limb of the syncline suggests they can only be explained by considering an external rotation. As implied from the above results, the dextral shear indicators originated during the initial phase of the oblique thrusting might be turned to the present sinistral position due to the formation of overturned drag fold in the footwall of the Moldanubian Thrust (Fig. 7c). The explanation of the sinistral strike slips in the MZ as dextral indicators overturned by external rotation perfectly fits the model of the large syncline because the overturning of kinematic indicators results in a seemingly opposite sense of movement.

## Methods

Analysis of the mesoscopic structures of the Brno Massif was performed to recognize large-scale structures that extend beyond the Brno Massif itself. Mesoscopic foliation is mostly defined by the preferred orientation of biotite in granodiorite (Fig. 3a) and by the cleavage planes in metabasite. Altogether 257 planar and linear features were obtained directly from outcrops, and these data were consequently processed using SpheriStat software, which allows for performing principal direction analysis including fold axis computation.

To verify, support, and extend the knowledge of mesoscopic data, anisotropy of magnetic susceptibility (AMS) was used. For AMS analysis, 1017 samples from 76 sites were analyzed using the KLY-5 Kappabridge by AGICO, Inc. which uses a 3D rotator^55^. Thereafter, the mesoscopic and magnetic foliations of the analyzed samples were compared and processed. Due to a straightforward relationship between the mesoscopic and AMS data (i.e., the parallelism between petrofabrics and AMS fabrics), the AMS tool was applied to compensate for missing data where no mesoscopic foliation could be easily identified in the field.

AMS is a petrophysical method appropriate for determining the magnetic susceptibility tensor **K**^56^. It can be visualized by an ellipsoid with three principal susceptibility directions, **K**_**max**_, **K**_**int**_, and **K**_**min**_. The lengths of the ellipsoid axes are the principal susceptibility values, *K*_*max*_, *K*_*int,*_ and *K*_*min*_. Magnetic lineation is parallel to the direction **K**_**max,**_ and the normal line to magnetic foliation is parallel to the direction **K**_**min**_. The values of these principal AMS directions serve as input for the calculation of AMS parameters. Here, we used three parameters for the description of the geometric features of the AMS ellipsoid^57^: (1) the mean magnetic susceptibility *K*_*mean*_ is given by the formula: *K*_*mean*_ = (*K*_*max*_ + *K*_*int*_ + *K*_*min*_)/3; (2) the anisotropy degree *P* reflects the intensity of the preferred orientation of magnetic minerals in a rock and is given by the following formula: *P* = *K*_*max*_/*K*_*min*_; (3) the shape parameter *T* reflects the oblateness or prolateness of the ellipsoid: *T* = 2 ln (*K*_*int*_ / *K*_*min*_*)* / ln (*K*_*max*_ / *K*_*min*_) – 1. An oblate magnetic ellipsoid representing rather a planar magnetic fabric takes positive *T* values (0 < *T* ≤ 1), while a prolate magnetic ellipsoid representing rather linear magnetic fabrics is defined by negative values of the shape parameter (0 > *T* ≥  − 1). All AMS data were processed with Anisoft 5.1 software (AGICO, Inc.). Consequently, the **K**_max_ and **K**_min_ directions were transferred from Anisoft 5.1 to SpheriStat 3.0 software as magnetic lineation and magnetic foliation poles, respectively, to perform the Gauss density distribution analysis on these data.

Furthermore, we analyzed the temperature dependence of magnetic susceptibility to recognize which minerals are the main magnetic carriers responsible for the AMS using the Kappabridge MFK-1 together with a CS4 furnace and CS-L cryostat apparatus (AGICO, Inc.) in the temperature range from − 192 to + 700 °C. The measurement was performed in a field of 200 A/m and a frequency of 976 Hz. Heating was conducted using powdered samples in an argon atmosphere with a heating rate of approximately 10 °C/min. The ferro/para resolution on the resulting curves was evaluated using a hyperbola fitting algorithm for temperature dependence curves following the Curie–Weiss law^58^.

The identification of dominant components of magnetic fabrics employed spatial averaging of oriented data with Orientation Analysis Tools by^59^. In this tool, the sum of orientation matrices is weighted by an inverse distance from the averaging station^60^. The interval of stations was set at 2000 m, and the influence radius of each station was defined at 8000 m. The magnetic parameters *P*, *T,* and *K*_*mean*_ at the sites were interpolated from the raster using weights of raster points based on proportionate areas to interpolate a value—the Natural Neighbor interpolation method^61^. AMS data were averaged for all sites and all types of rock at the sites. The shape parameter *T* was separated for linear (*T* <  − 0.05), planar (*T* > 0.05), and triaxial fabrics (*T* ranging from − 0.05 to 0.05). The anisotropy degree was divided into three groups representing sites with low, intermediate, and high anisotropies.

## Supplementary Information

Supplementary Information 1.

Supplementary Information 2.

## Data Availability

The magnetic anisotropy data are provided in Supplementary material as RAN files readable in freeware Anisoft by AGICO company (compressed in a single ZIP file named SupDat_AMS.zip).
