# Supplementary material for: Non-coaxial deformation of foreland basement involved in a fold-and-thrust belt: a strain partitioning approach to the Eastern Variscan orogen
Source: Sci Rep. 2023 May 19;13:8143. doi: 10.1038/s41598-023-35400-4 (PMC10199064; doi:10.1038/s41598-023-35400-4)
Supplement: Supplementary file 1 — Supplementary Information 1. [file 41598_2023_35400_MOESM1_ESM.pdf]

Supplementary information

Non-coaxial deformation of foreland basement involved in a fold-and-thrust belt: a strain partitioning approach to the Eastern Variscan orogen

***L. Mareček[a], R. Melichar[a], J. Černý[a, b, c], P. Schnabl[b], K. Hrdličková[d], D. Buriánek[d]**

[a] Department of Geological Sciences, Faculty of Science, Masaryk University, Brno, Czech Republic

[b] Institute of Geology of the Czech Academy of Sciences, Rozvojová 269, 165 00 Prague-Lysolaje, Czech Republic

[c] Helmholtz-Zentrum Dresden-Rossendorf, Helmholtz-Institute Freiberg for Resource Technology, Freiberg, Germany

[d] Czech Geological Survey, Klárov 3, 118 21 Prague 1, Czech Republic

e-mail addresses: Lukáš Mareček (marecekluk@gmail.com; +420720372519)

Rostislav Melichar (melda@sci.muni.cz)

Jan Černý (17.sci.geo@gmail.com)

Petr Schnabl (schnabl@gli.cas.cz)

Kristýna Hrdličková (kristyna.hrdlickova@geology.cz)

David Buriánek (david.burianek@geology.cz)

Oblique thrusting orogens in the world

Collisional orogenic belts are strongly thrust-folded [1] as a result of the convergent movements of continental tectonic plates. According to the direction of convergence, two principal cases of structural models can be applied to current orogens: (1) frontal thrusting orogens, which are typified by a thrusting direction that is more or less perpendicular to the orogenic frontal zone (e.g., [1, 2]), and (2) oblique thrusting orogens, which originate when the direction of thrust movement is oblique to, or even almost parallel to, the frontal line [3, 4]. Oblique convergence must naturally occur whenever an orogenic belt is bent, whereas the direction of convergence movement remains the same. Such oblique collisions are not uncommon and have been described from a variety of old and young orogens, such as the Precambrian orogen in the southern Brazilian Craton [5], the Grenville orogen in Canada [6], the Variscan orogen in Iberia [7], and the recent Alpine–Himalayan orogens in Zagros [8], the Himalayan Tibetan plateau [9], and the southeastern Alps [10]. Exemplary oblique thrusting has been recognized in the easternmost part of the Central European Variscan Zone [11, 12, 13, 14, 15], where the course of the various Variscan zones turns sharply from the E-W direction to the N-S direction, while the collisional orientation still remains in the N–NNE direction (Fig. 1a).

Brief insight to tectonic history of the eastern margin of Central European Variscides

The Variscan collision on the easternmost margin of the Central European Variscides starts with the convergence of the Lugodanubian plate and the Brunovistulicum. The first signs of compression are shown by the transition from quiet Devonian carbonate sedimentation to Carboniferous flysch clastic sedimentation. At the end phase of this clastic sedimentation, the

pebbles containing material from the Lugodanubian unit were deposited in the flysch complex, proving uplift and exhumation of Lugodanubian block above the Moldanubian thrust. Folded sedimentary rocks of the Moscovian age date the last movements of the oblique thrusting. Then, the oblique thrusting turns into a dextral strike slip movement evidenced by a mylonite zone associated with the marginal fault of the future Boskovice half-graben. In the Boskovice half-graben, sedimentation began in the latest Gzhelian (Late Carboniferous). This marks the beginning of the period of the gravitational collapse of the eastern Variscan orogen.

Supplementary magnetomery in rocks of the Brno Massif

The temperature dependence of magnetic susceptibility in the analyzed rocks from the Brno Massif was supplemented by the isothermal remanent magnetization (IRM) and the field dependence of the susceptibility studies. IRM was acquired using a combination of the AGICO instrument LDA-5/PAM-1 and the Magnetic Measurements Pulse Magnetizer MMPM10 in the field range of 2 to 2000 mT and measured on an AGICO JR6 spinner magnetometer. The coercivity spectra were quantified by the gradient acquisition plot method using MAX UNMIX software by Dan Maxbauer based on mathematics published by [16].

Statistical analysis was conducted after [17, 18]. The obtained magnetic components are characterized by three parameters: (1) saturation magnetization (SIRM); (2) mean coercivity ($B_{1/2}$), which reflects the applied magnetic field (B) at which half of the SIRM is reached; and (3) dispersion parameter (DP) of the cumulative log-normal distribution. The mineralogy of the components was evaluated following the publications by [19, 20]

The field dependence of magnetic susceptibility (k_{HD}) was measured in the field range of 5 to 700 A/m and the frequency of 976 Hz using an AGICO Kappabridge MFK-1 instrument. The field dependence was quantitatively evaluated as follows.

$$k_{HD}\ [\%] = 100 \times (k_{300} - k_{30}) / k_{300}$$

where k_{30} and k_{300} are the susceptibilities measured in the magnetic field with the amplitude expressed in A/m (*sensu* [21]).

IRM results

The IRM decomposition results (Supplementary Fig S1 and Table S1) prove four components: component 1 has $B_{1/2}$ around 30 mT and corresponds to multidomain magnetite; component 2 (62–133 mT) corresponds to single domain magnetite; component 3 (312–882 mT) corresponds to high coercivity iron oxide(hydroxide), and component 4 with $B_{1/2}$ more than 2T corresponds to goethite. Field-dependent magnetic susceptibility of around 0 % proves chemically pure magnetite without Ti or Al admixtures [22].

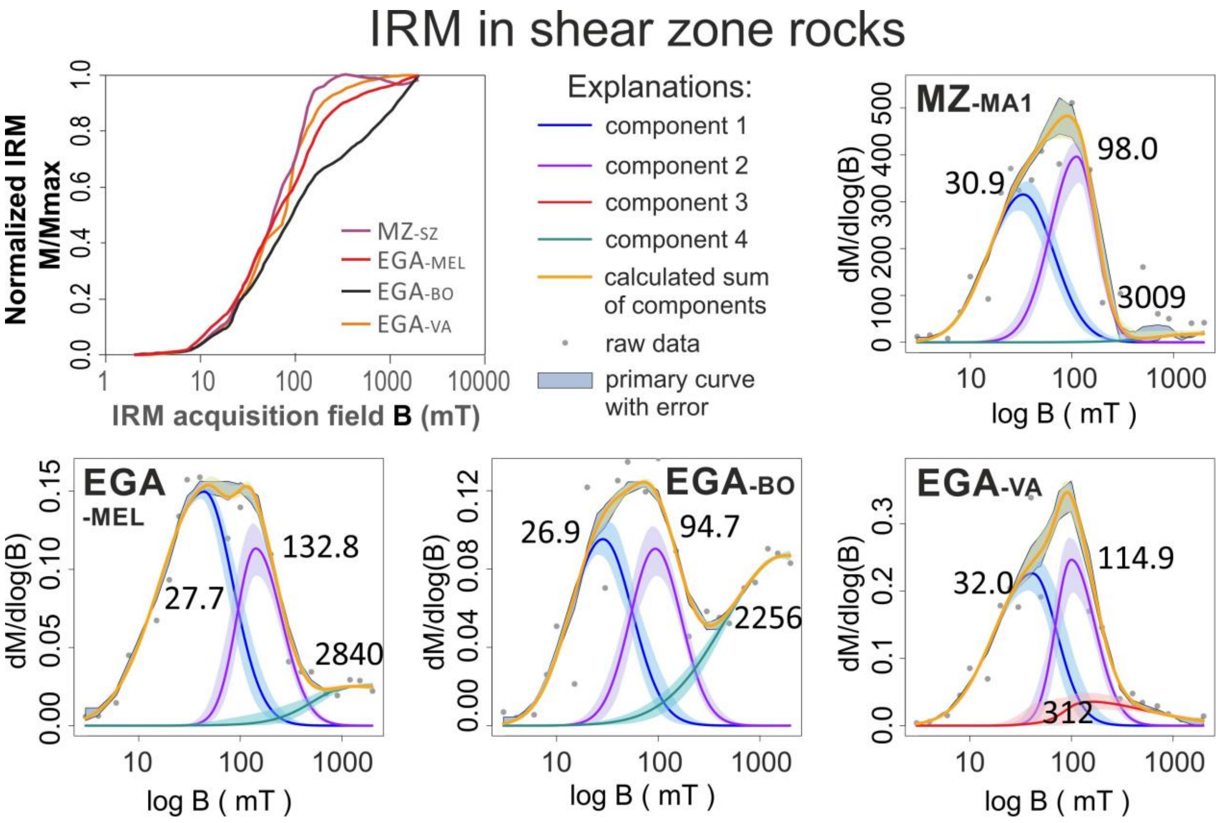

Figure S1: The IRM acquisition curves (top left) and component analyzes of the IRM curves (top right and bottom) were conducted following [4, 20]. Belts around curves represent estimated errors and the IRM gradient is defined as dM/dlog(B), where M is magnetization and B is a magnetic field.

locality	component 1	component 2	component 3	component 4	k_{HD}
MZ-MA1	30.9 / 52	98.0 / 46	-	3009 / 2	-0.85
EGU-MEL	27.7 / 53	132.8 / 38	-	2840 / 9	5.48
EGU-BO	26.9 / 36	94.7 / 29	-	2256 / 35	4.37
EGU-VA	32.0 / 53	114.9 / 36	312 / 11	-	3.35

Table S1: Four components calculated during IRM decomposition and field dependence (k_{HD}) results. Numbers separated by slashes represent the mean coercivity $B_{1/2}$ [mT] and the relative amount of the ferromagnetic component [%]. Bear in mind that IRM does not provide information on paramagnetic phases and their contributions.

Distribution of sites and its average orientation

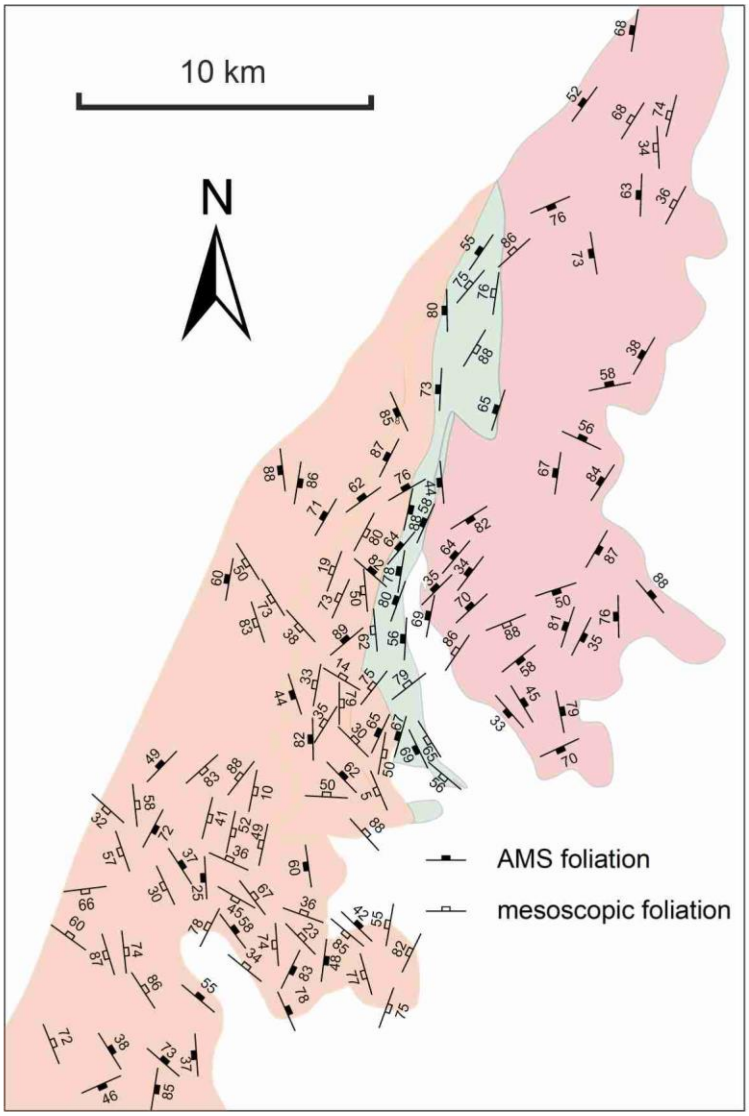

Fig.S2: The distribution of AMS and mesoscopic foliations of individual sites in a schematic map of the Brno Massif (modified after [23]. The figure was created using ArcGIS 10.2 software (URL: https://www.esri.com/en-us/arcgis/products/arcgis-desktop/overview).

Distribution of mesoscopic and AMS data in zones of Brno Massif

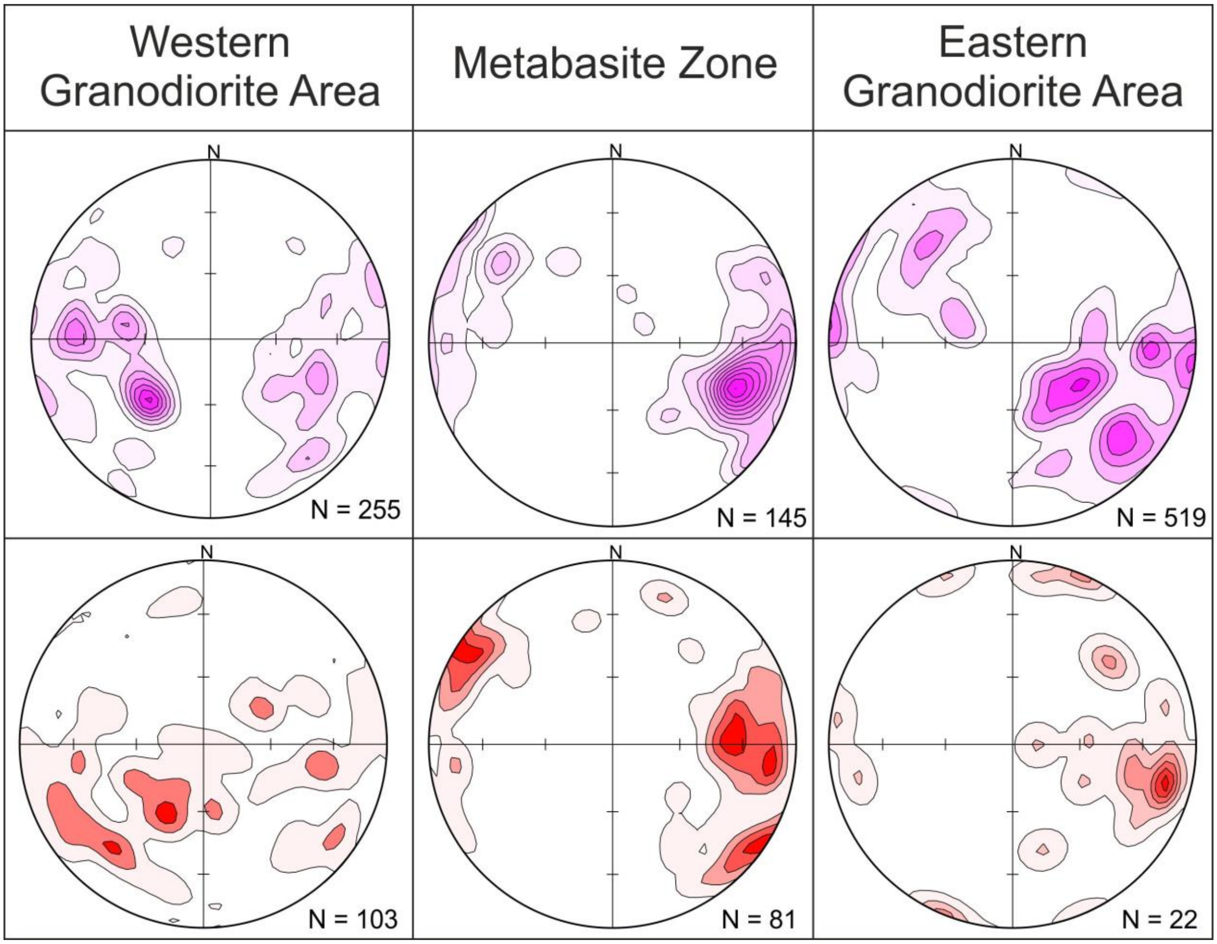

Fig. S3: Orientation of magnetic (upper row) and mesoscopic (lower row) foliations for individual zones of the Brno Massif from west to east. Diagrams are in equal-area Schmidt projection on lower hemisphere, contour lines with interval 2× sigma.

References:

1. Rosenberg, C. & Kissling, E. Three-dimensional insight into Central-Alpine collision: Lower-plate or upper-plate indentation? Geology 41, 12, 1219-1222, (2013).
2. Gansser, A. Geology of the Bhutan Himalaya. Basel: Birkhauser Verlag. ISBN 3 7643 1371 4, (1983).
3. McClay, K., R., Whitehouse, P., S., Dooley, T. & Richards, M. 3D evolution of fold and thrust belts formed by oblique convergence. Marine and Petroleum Geology, Volume 21, Issue 7, pages 857-877, ISSN 0264-8172, https://doi.org/10.1016/j.marpetgeo.2004.03.009, (2004).

4. Bajolet, F., Replumaz, A. & Lainé, R. Orocline and syntaxes formation during subduction and collision. Tectonics, 32(5), 1529–1546. https://doi.org/10.1002/tect.20087, (2013).
5. Ebert, H. D. & Hasui, Y. Transpressional tectonics and strain partitioning during oblique collision between three plates in the Precambrian of southeast Brazil. Geological Society, London, Special Publications, 135(1), 231–252. https://doi.org/10.1144/gsl.sp.1998.135.01.15, (1998).
6. Sharma, K. N. M., Singhroy, V. H. & Lévesque, J. A two-stage emplacement for the Cabonga allochthon (central part of the Grenville Province): evidence for orthogonal and oblique collision during the Grenville orogeny. Canadian Journal of Earth Sciences, 32(9), 1474–1478. https://doi.org/10.1139/e95-119, (1995).
7. Corsini, M., & Rolland, Y. Late evolution of the southern European Variscan belt: Exhumation of the lower crust in a context of oblique convergence. Comptes Rendus Geoscience, 341(2–3), 214–223. https://doi.org/10.1016/j.crte.2008.12.002, (2009).
8. Authemayou, C., Chardon, D., Bellier, O., Malekzadeh, Z., Shabanian, E. & Abbassi, M. R. Late Cenozoic partitioning of oblique plate convergence in the Zagros fold-and-thrust belt (Iran). Tectonics, 25(3), https://doi.org/10.1029/2005tc001860,(2006).
9. Allen, M. B., Walters, R. J., Song, S., Saville, C., De Paola, N., Ford, J., Hu, Z. & Sun, W. Partitioning of oblique convergence coupled to the fault locking behavior of fold-and-thrust belts: Evidence from the Qilian Shan, northeastern Tibetan Plateau. Tectonics, 36(9), 1679–1698. https://doi.org/10.1002/2017tc004476, (2017).
10. Polinski, R. K. & Eisbacher, G. H., 1992. Deformation partitioning during polyphase oblique convergence in the Karawanken Mountains, southeastern Alps. Journal of Structural Geology, 14(10), 1203–1213. https://doi.org/10.1016/0191-8141(92)90070-d
11. Grygar, R. Kinematics of Lugosilesian Orocline accretion wedge in relation to the Brunovistulian foreland. Sborník vědeckých prací Vysoké školy báňské v Ostravě. Řada hornicko-geologická, 38(1), 49-72. ISSN 0474-8476, (1992).
12. Kolaříková, A., Marquer, D. & Schulmann, K. Evolution of mass-transfer during progressive oblique under-thrusting of the Variscan foreland: eastern Bohemian Massif. Geodinamica Acta, 10(3), 81–93. https://doi.org/10.1080/09853111.1997.11105295, (1997).
13. Schulmann, K. & Gayer, R. A model for a continental accretionary wedge developed by oblique collision: the NE Bohemian Massif. Journal of the Geological Society, 157(2), 401–416. https://doi.org/10.1144/jgs.157.2.401, (2000).
14. Tomek, F., Vacek, F., Žák, J., Petronis, M.S., Verner, K. & Foucher, M.S., Polykinematic foreland basins initiated during orthogonal convergence and terminated by orogen-oblique strike-slip faulting: An example from the northeastern Variscan belt. Tectonophysics, 766: 379-397, (2019).
15. Mazur, S., Aleksandrowski, P., Gągała, U., Krzywiec, P., Żaba, J., Gaidzik, K., & Sikora, R. Late Palaeozoic strike-slip tectonics versus oroclinal bending at the SW outskirts of Baltica: case of the Variscan belt's eastern end in Poland. International Journal of Earth Sciences, 109(4), 1133–1160. https://doi.org/10.1007/s00531-019-01814-7, (2020).

16. Egli, R. Analysis of the field dependence of remanent magnetization curves. *Journal of Geophysical Research: Solid Earth*, **108**(B2). https://doi.org/10.1029/2002jb002023, (2003).

17. Kruiver, P. P., Dekkers, M. J., & Heslop, D. Quantification of magnetic coercivity components by the analysis of acquisition curves of isothermal remanent magnetisation. *Earth and Planetary Science Letters*, **189**(3–4), 269–276. https://doi.org/10.1016/s0012-821x(01)00367-3, (2001).

18. Heslop, D., Dekkers, M. J., Kruiver, P. P., & Van Oorschot, I. H. M. Analysis of isothermal remanent magnetization acquisition curves using the expectation-maximization algorithm. *Geophysical Journal International*, **148**(1), 58–64. https://doi.org/10.1046/j.0956-540x.2001.01558.x, (2002).

19. Dudzisz, K., Kontny, A., & Alva-Valdivia, L. M. Curie Temperatures and Emplacement Conditions of Pyroclastic Deposits From Popocatépetl Volcano, Mexico. *Geochemistry, Geophysics, Geosystems*, **23**(8). https://doi.org/10.1029/2022gc010340, (2022).

20. Huang, W., et al. Can a primary remanence be retrieved from partially remagnetized Eocence volcanic rocks in the Nanmulin Basin (southern Tibet) to date the India-Asia collision? *Journal of Geophysical Research: Solid Earth*, **120**(1), 42–66. https://doi.org/10.1002/2014jb011599, (2015).

21. Vahle, C., Kontny, A., Gunnlaugsson, H., & Kristjansson, L. The Stardalur magnetic anomaly revisited—New insights into a complex cooling and alteration history. *Physics of the Earth and Planetary Interiors*, **164**(3–4), 119–141. https://doi.org/10.1016/j.pepi.2007.06.004, (2007).

22. Schnabl P., *et al.* Magnetic properties of high-Ti basaltic rocks from the Krušné hory/Erzgebirge MTS. (Bohemia/Saxony), and their relation to mineral chemistry, *Stud. Geoph. Et Geodet.*, **54**, 1, 77 – 94, (2010).

23. Cháb, J., Stráník, Z. & Eliáš, M. Geological map of the Czech Republic 1 : 500,000. Czech Geological Survey. Praha, (2007).
